# Association between metabolic syndrome and colorectal cancer incidence and all-cause mortality: a hospital-based observational study

**DOI:** 10.1186/s12876-022-02505-5

**Published:** 2022-11-11

**Authors:** Kuan-Chih Chung, Sin-Ei Juang, Hong-Hwa Chen, Kung-Chuan Cheng, Kuen-Lin Wu, Ling-Chiao Song, Ko-Chao Lee

**Affiliations:** 1 Department of Anesthesiology, Kaohsiung Chang Gung Memorial Hospital, Chang Gung University College of Medicine, Kaohsiung, Taiwan; 2 Division of Colorectal Surgery, Department of Surgery, Kaohsiung Chang Gung Memorial Hospital, Chang Gung University College of Medicine, Kaohsiung, Taiwan; 3 Division of Colon & Rectal Surgery, Department of Surgery, E-DA Hospital, I-Shou University, Kaohsiung, Taiwan

**Keywords:** Metabolic syndrome (MetS), Colorectal cancer (CRC), Mortality

## Abstract

**Background:**

Metabolic syndrome (MetS) is a worldwide pandemic and complex disorder associated with colorectal cancer (CRC). This study aims to identify the influence of number of MetS components on CRC incidence and mortality, using a national, longitudinal dataset of hospital care in Taiwan.

**Methods:**

Patient data from the Taiwan National Health Insurance Research Database (NHIRD) from 2001 to 2008 were extracted. Individuals with at least one inpatient diagnosis or 2 outpatient visits with any MetS component found within one year were identified and included. Subjects died within 12 months after the presence of MetS components or had any prior cancer were excluded. The study cohort were then divided into two groups: subjects who had more (i.e., 3 to 4) MetS components and those who had fewer (i.e., 1 to 2) MetS components. An 2:1 propensity score (PS) matching were performed to balance the baseline characteristics between the groups. Cox regression analyses were conducted to compare the CRC incidence and all-cause mortality at follow-up between subjects with more MetS components versus fewer components.

**Results:**

After matching, a total of 119,843 subjects (78,274 with 1–2 and 41,569 with 3–4 MetS components) were analyzed. After adjusting for confounders, subjects with 3–4 MetS components had a significantly higher risk of CRC [adjusted hazard ratio (aHR), 1.28; 95% confidence interval (CI), 1.15–1.43, p < 0.001) and all-cause mortality (aHR, 1.13; 95% CI, 1.08–1.17, p < 0.001) than those with only 1–2 MetS components. In stratified analyses, the greatest increased risk of CRC incidence that 3–4 MetS components posed as compared to 1–2 MetS components was seen in subjects without CHD history (aHR, 1.41, 95% CI, 1.23–1.62, p < 0.001). In addition, 3–4 MetS components (vs. 1–2) led to greater all-cause mortality among the subjects < 65y, both genders, with or without CHD, subjects without CKD hisotry, both aspirin users and non-users, users of nonsteroidal anti-inflammatory drugs (NSAIDs), and users of statin.

**Conclusion:**

Compared with 1–2 components, subjects with 3–4 MetS components are at greater risk of CRC and death at follow-up. This study also demonstrates the risks for CRC and all-cause mortality in certain subgroups of individuals with 3–4 MetS components compared to 1–2 components. These findings may help clinicians on the CRC risk stratification according to individuals’ characteristics, as well as to optimize the strategy of MetS surveillance and control in order to prevent CRC.

## Background

Metabolic syndrome (MetS) is a worldwide pandemic and complex disorder, defined as a combination of interconnected factors which caused metabolic, anthropometric, and hemodynamic abnormalities [1, 2]. It is directly associated with the risks for coronary heart disease (CHD), cardiovascular diseases (CVD), and type 2 diabetes mellitus (T2DM) [2, 3]. The global prevalence of MetS is still rising now, with an increasing trend observed in children and young adults specifically, posing a significant public health burden [4–6]. Importantly, according to the medical literature to date, MetS has been linked to the development of certain cancers and cancer-associated deaths [1, 7, 8].

Notably, colorectal cancer (CRC) is one of the MetS-associated cancers [9, 10]. The global burden of CRC was expected to increase by 60% by 2030, including not only larger case number but also more deaths [9]. Lifestyle factors, such as poor dietary habits (low fruit and vegetables consumption and high intake of red/processed meats), sedentary behavior, or cigarette smoking are indicated to be responsible for the uprising trend of CRC [11–14].

Although some evidence suggested MetS is a risk factor for CRC and subsequent death thereof [1, 4, 8, 15–18], other researchers were not sure about the precise role of MetS on CRC development [19, 20]. Further, although an individual who met the criteria of MetS were at greater risk of CRC, no comparison has been performed on the risk of CRC between subjects with 3 to 4 MetS components versus those who had only 1 to 2 MetS components. Given the burden of both MetS and CRC pose to the society and the healthcare system, it is of special importance to gain in-depth understanding about the role of MetS on CRC risk in view of number of MetS components.

Following the context, therefore, the present study aimed to compare the incidence of CRC and mortality between individuals with 3 to 4 MetS components and 1 to 2 components, using a large national, longitudinal dataset of hospital care. We hypothesized that subjects with 3 to 4 MetS components have a significantly greater CRC incidence and higher all-cause mortality than those who had 1 to 2 MetS components only.

## Methods

### Data source

Subjects’ data were all extracted from the National Health Insurance Research Database (NHIRD), a part of the National Health Insurance (NHI) system of Taiwan. The NHI system of Taiwan is a nationwide insurance system established in 1995 which covers 99% of 23.74 million people in Taiwan [21]. Based on claim data, NHIRD collects both outpatient and inpatient information which begins at the year 2000 [21]. In particular, NHIRD contains healthcare information of individuals during hospital admissions, outpatient visits, including diagnoses, orders and procedures performed. Diagnoses are coded through the International Classification of Diseases, 9th Revision of Clinical Modification (ICD-9-CM) system.

The present study was approved by the Research Ethics Review Committee of the Chang Gung Memorial Hospital. Requirement for informed consent was waived due to all information in this dataset were anonymized and de-identified.

### Study population

In the present study, data of subjects aged at and above 40 years and with new presence of any MetS from 2001 to 2008 in the NHIRD were retrieved, and were lagged for one year. The inclusion criteria were: 1) at least one inpatient or two outpatient diagnoses with any components of MetS found within one year. The lag time design (i.e., the index date was defined as 1 year after the last component of MetS was diagnosed) was imposed to account for the latency period in CRC development. The exclusion criteria were: (1) subjects who died within 12 months after the presence of MetS components; (2) subjects who had been diagnosed with any cancers (ICD-9-CM: 140–239) before the presence of MetS components.

**Fig. 1:**
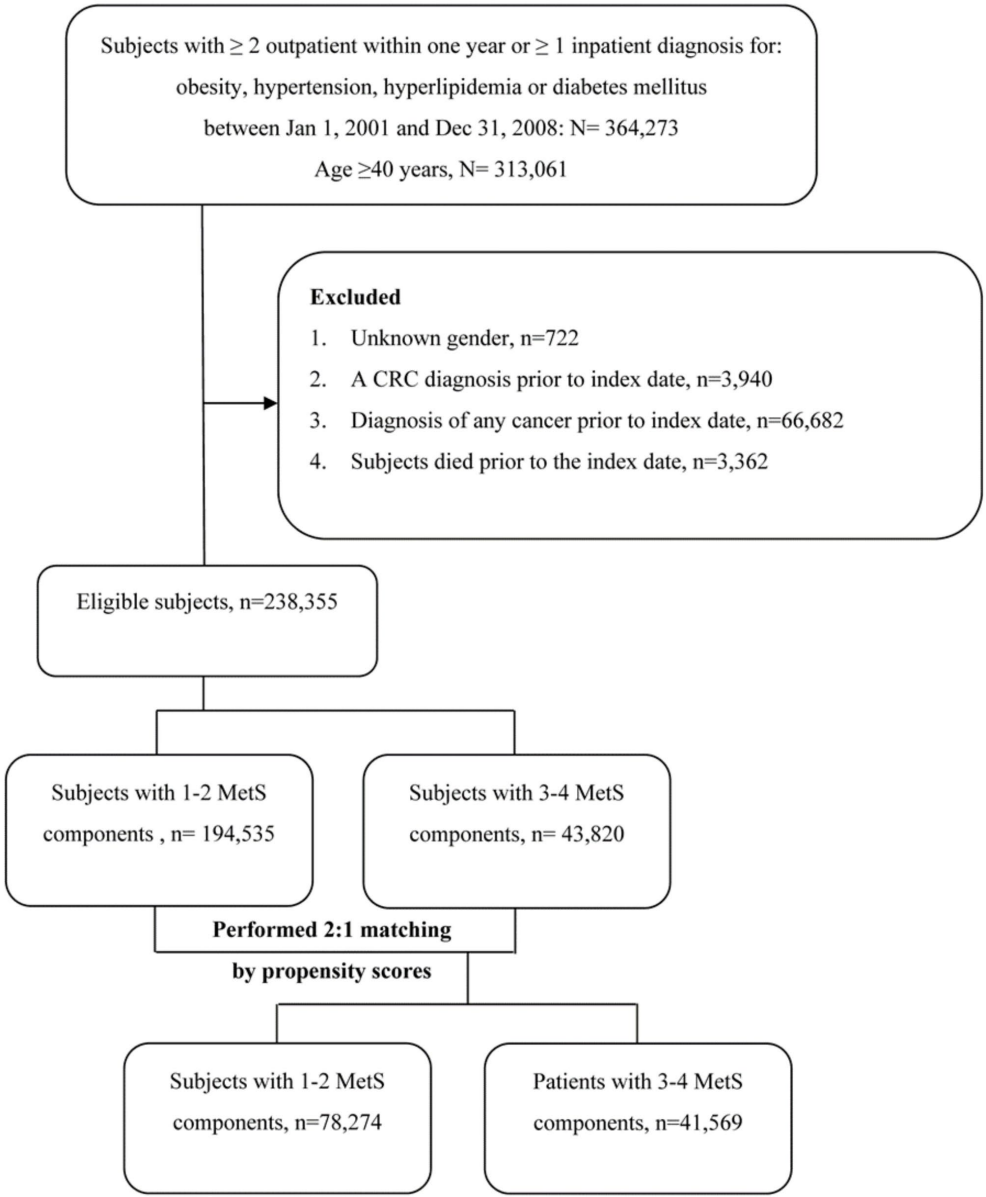
Flow chart of study selection process

The selected cohort was then divided into two comparison groups: subjects with 3–4 MetS components and subjects with only 1–2 MetS components. A 2:1 propensity score (PS) matching was performed to balance the baseline characteristics between the two groups. Multivariable logistic regression was adjusted for the following relevant covariates; age, sex, index year, smoking, alcohol drinking, comorbidities, renal transplantation, and medications prescribed. Figure 1 shows the selection process of the study population.

### Study variables

#### Definition of metabolic syndrome (MetS)

According to the National Cholesterol Education Program Adult Treatment Panel III, the MetS status was defined based on the presence of any three or more of the following criteria:

(1) Abdominal obesity, i.e., an elevated waist circumference > 102 cm for men and > 88 cm for women. Since the NHIRD dataset lacks waist circumference measures, we utilized the diagnosis of obesity (i.e., a BMI ≥ 30 kg/m^2^; ICD-9-CM: 278.0) as a surrogate in accordance with a previous claim-based study [22].

(2) Elevated triglycerides (≥ 150 mg/dL), identified through usage of lipid-lowering drug, or a diagnosis of hyperlipidemia (ICD-9-CM: 272);

(3) Reduced high-density lipoprotein cholesterol (HDL-c) (i.e., < 40 mg/dL in men and < 50 mg/dL in women). We used the diagnosis of hyperlipidemia (ICD-9-CM: 272) to surrogate this component.

(4) Elevated blood pressure (i.e., SBP ≥ 130 mmHg or DBP ≥ 85 mmHg), identified through usage of antihypertensive medications or a diagnosis of hypertension (ICD-9-CM: 401 or 405); and.

(5) Elevated fasting blood glucose (i.e., ≥ 100 mg/dL), identified through usage of antidiabetic medications or a diagnosis of diabetes mellitus (DM) (ICD-9-CM: 250).

#### Study endopoints

The primary endpoint was the incidence of CRC (ICD-9-CM: 153–154), identified through one inpatient diagnosis or two outpatient diagnosis found after the presence of MetS components. The secondary endpoint was all-cause mortality.

#### Covariates

Demographic variables included age and gender. Cigarette smoking (ICD-9-CM: 305.1), alcohol drinking (ICD-9-CM: 291, 303, 305.0, 357.5, 425.5, 535.3, and E860.0), and comorbidities including liver disease (ICD-9-CM: 571), inflammatory bowel disorders (ICD-9-CM: 555, 556), CHD (ICD-9-CM: 410–414), Helicobacter pylori infection (ICD-9-CM: 041.86), chronic kidney disease (CKD) (ICD-9-CM: 585), and renal transplantation (ICD_OP_CODE = 55.6, ORDER_CODE = 76,020 A, 76020B, 97,416 K, 97,417 A, 97418B) were included as covariates. Medications prescribed, such as aspirin (ATC codes: B01AC06, N02BA01, N02BA51), non-steroidal anti-inflammatory drugs (NSAIDs) (ATC group: M01A), statins (ATC group: C10AA, C10BA, C10BX03), and sex hormones/endocrine therapy (ATC codes: L02 and G03) were also included in the analysis.

### Statistical analysis

Pairwise analyses were conducted to check the differences between subjects with 1–2 and 3–4 MetS components. PS matching was conducted to balance the baseline characteristics between the groups. Multivariable logistic regression was used to compute the propensity score using the following baseline covariates: age, sex, index year, smoking, alcohol drinking, comorbidities, renal transplantation and concurrent medications. Greedy nearest-neighbor matching on the PS with the width of 0.03 was performed [23]. Baseline characteristics were compared between groups using standardized differences with weighted proportions to properly account for the matched nature of the sample [24]. After matching, Cox regression models were used to compare the risk of CRC and all-cause mortality between the groups. The assumption of proportional hazards was checked, and the robust estimation method was used to account for the clustering within matched sets [25]. The Kaplan-Meier method was used to estimate the probability of CRC-free and overall survival.

Stratified analyses (by age, sex, history of CHD and CKD, use of aspirin, non-aspirin NSAIDs, or statins) were conducted. The adjusted hazard ratios (aHRs) were derived. Interactions between each stratification factor and number of MetS components were tested. Two-sided p-value < 0.05 was consider as a significant result. Data management and statistical analyses were conducted using SAS version 9.4 software (SAS Institute, Inc.).

## Results

### Baseline characteristics of the study cohort

**Fig. 2:**
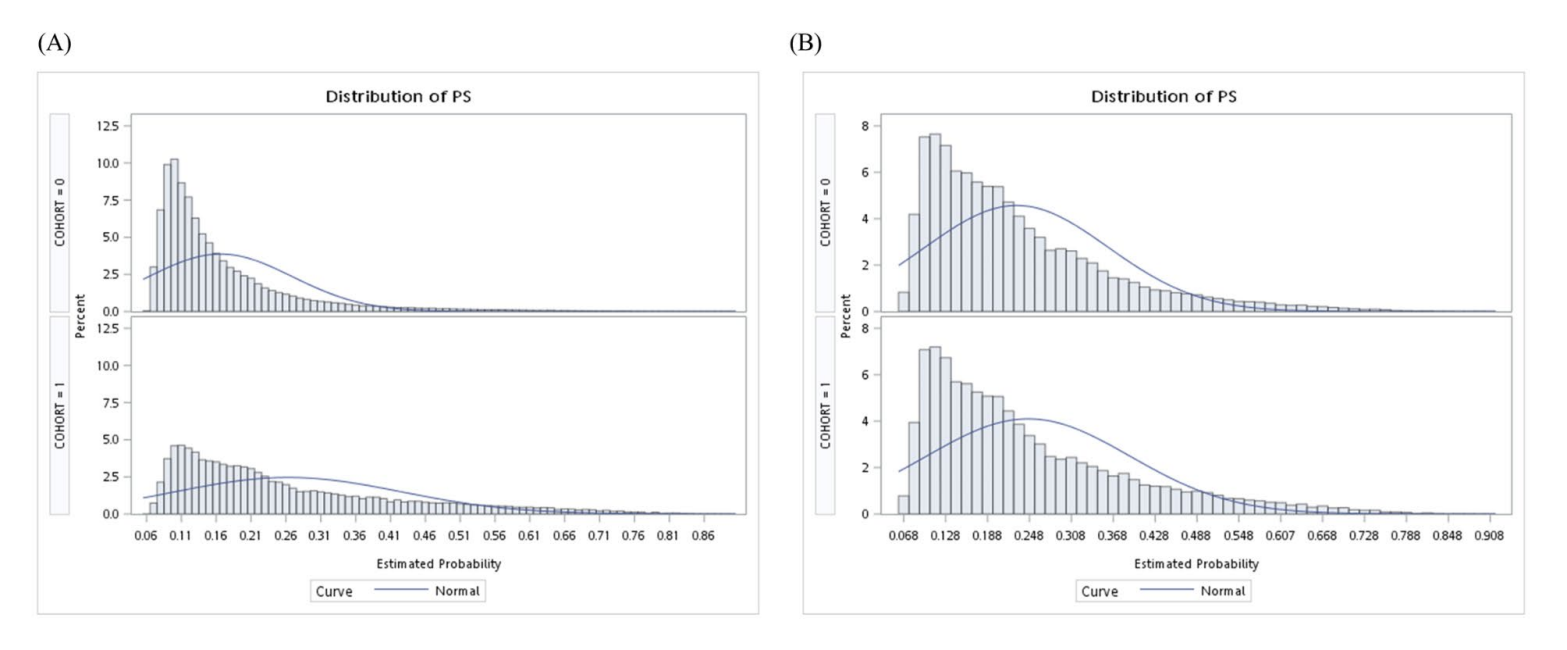
Propensity score distribution in subjects with 1–2 MetS components (cohort = 0) or 3–4 MetS components (cohort = 1). (A) Before matching. (B) After matching

A total of 238,355 subjects who met the inclusion criteria were enrolled initially. There were 194,535 subjects with 1–2 MetS and 43,820 subjects and 3–4 components of MetS being identified. After 2:1 PS matching, 78,274 and 41,569 subjects with 1–2 and 3–4 MetS components were included as the primary cohort. All baseline characteristics were well-balanced with absolute standardized differences < 0.1 after matching (Table 1). The PS distributions before and after matching are shown in Fig. 2.

**Table 1:** Baseline characteristics of study population grouped by number of MetS components (after 2:1 PS matching)

Characteristics	1–2 components (N = 78,274)	3–4 components (N = 41,569)	Std Diff
Age, mean ± SD	61.73 ± 11.68	61.65 ± 11.00	-0.03
Male sex	39,474 (50.43%)	20,507 (49.33%)	-0.02
Index year			0.01
2002–2003	16,592 (21.2%)	8647 (20.8%)	
2004–2005	20,120 (25.7%)	10,634 (25.58%)	
2006–2007	20,990 (26.82%)	11,328 (27.25%)	
2008–2009	20,572 (26.28%)	10,960 (26.37%)	
Smoking	413 (0.53%)	264 (0.64%)	0.01
Alcohol drinking	410 (0.52%)	279 (0.67%)	0.02
Comorbidity			
Liver disease	18,538 (23.68%)	10,401 (25.02%)	0.01
CHD	26,946 (34.43%)	14,053 (33.81%)	-0.05
H. pylori infection	119 (0.15%)	82 (0.20%)	0.01
CKD	2572 (3.29%)	1530 (3.68%)	0.01
Renal transplantation	9 (0.01%)	6 (0.01%)	0.001
Number of prescribed medications			
Aspirin			0.01
0–1	55,047 (70.33%)	28,235 (67.92%)	
2–5	8556 (10.93%)	4593 (11.05%)	
6–10	6353 (8.12%)	3557 (8.56%)	
≥11	8318 (10.63%)	5184 (12.47%)	
Non-aspirin NSAID			0.05
0–1	28,882 (36.90%)	14,126 (33.98%)	
2–5	17,541 (22.41%)	9531 (22.93%)	
6–10	11,679 (14.92%)	6547 (15.75%)	
≥11	20,172 (25.77%)	11,365 (27.34%)	
Statins			0.05
0–1	55,090 (70.38%)	27,520 (66.2%)	
2–5	13,758 (17.58%)	7181 (17.27%)	
6–10	6902 (8.82%)	4541 (10.92%)	
≥11	2524 (3.22%)	2327 (5.6%)	
Sex hormones/endocrine therapy			0.03
0–1	74,406 (95.06%)	39,246 (94.41%)	
2–5	1903 (2.43%)	1177 (2.83%)	
6–10	897 (1.15%)	525 (1.26%)	
≥11	1068 (1.36%)	621 (1.49%)	
Data are presented as mean ± SD or n (%). Abbreviations: CHD, coronary heart disease; CKD, chronic kidney disease; NSAID, nonsteroidal anti-inflammatory drugs; Std Diff, standardized difference.			

### CRC incidence and all-cause mortality

The median follow-up duration of subjects with 1–2 and 3–4 components were 4.39 and 4.35 years, respectively. Subjects with 3–4 components of MetS had a significantly higher event rates and risk for CRC than those with 1–2 components of MetS (280.85 vs. 223.09 per 100,000 person-years; crude HR, 1.26; 95% CI, 1.13–1.41, p-value < 0.001). Similarly, subjects with 3–4 components of MetS were also more likely to experience all-cause mortality than those with 1–2 components of MetS (2094.32 vs. 1900.11 per 100,000 person-years, HR, 1.10; 95% CI, 1.06–1.15, p-value < 0.001) (Table 2).

**Table 2:** CRC incidence and all-cause mortality in patients with 3–4 MetS components versus 1–2 MetS components

Outcomes	No.	Events	Follow-up duration (years)^a^	Event Rate (/100,000 Person-Years)	Crude HR (95% CI)	*P*-value
**CRC**						
1–2 components	78,274	792	4.39 (2.61–6.29)	223.09	Reference	**< 0.001**
3–4 components	41,569	526	4.35 (2.62–6.23)	280.85	**1.26 (1.13–1.41)**	
**All-cause mortality**						
1–2 components	78,274	6,781	4.42 (2.64–6.32)	1900.11	Reference	
3–4 components	41,569	3,947	4.39 (2.65–6.26)	2094.32	**1.10 (1.06–1.15)**	**< 0.001**
All comparisons were based on matched cohorts. Significant values are showing in bold. ^a^ Presented in medium (Q1-Q3).						
Abbreviations: CRC, colorectal cancer; HR, hazard ratio.						

### Adjusted risk for CRC and all-cause mortality

**Fig. 3:**
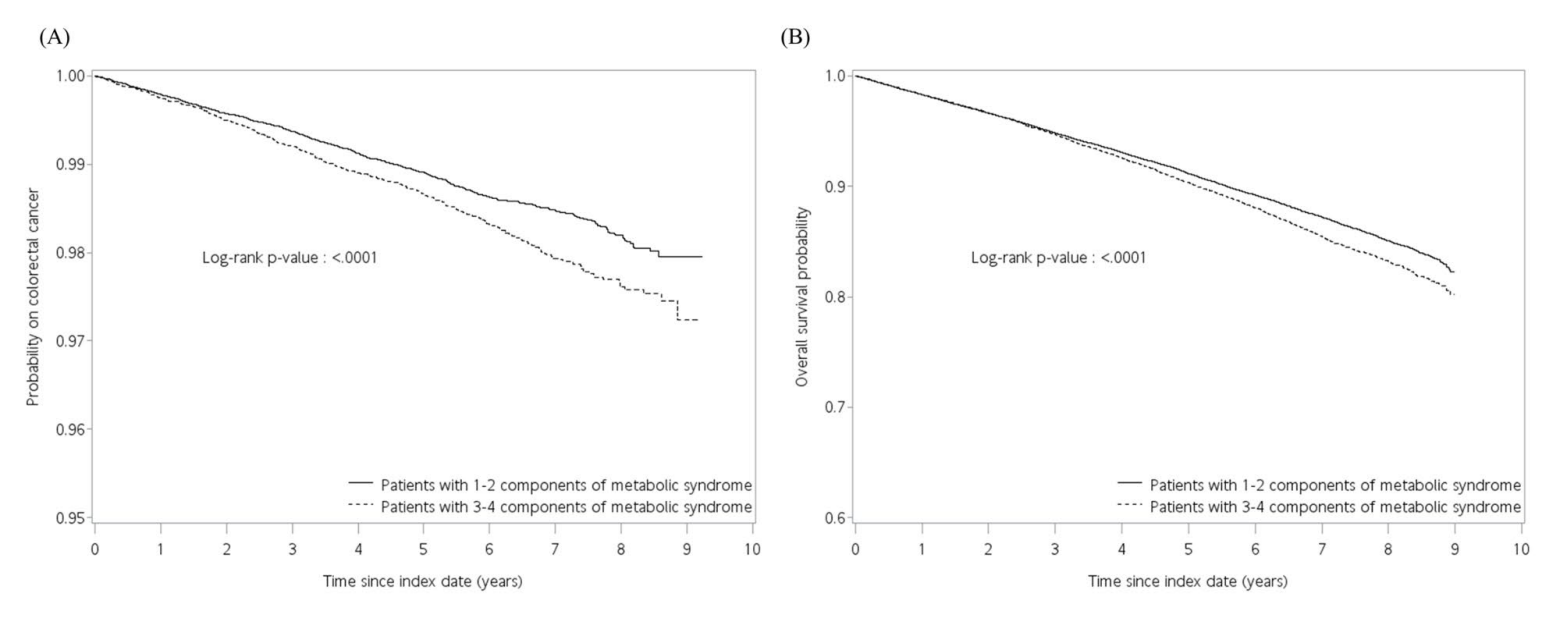
Kaplan-Meier curves for time to (A) CRC and (B) all-cause mortality in subjects with 1–2 or 3–4 MetS components (in matched cohort)

After adjusted for age, sex, index year, smoking, alcohol drinking, comorbidities, renal transplantation and medications prescribed, patients with 3–4 MetS components still had a higher risk for CRC and all-cause mortality (aHR, 1.28; 95% CI, 1.15–1.43, and aHR, 1.13; 95% CI, 1.08–1.17, respectively, both p-value < 0.001) (Table 3). The Kaplan-Meier curves for time to CRC and all-cause mortality in patients with 1–2 or 3–4 components of MetS are shown in Fig. 3.

**Table 3:** Adjusted and stratified CRC incidence and all-cause mortality in patients with 3–4 MetS components versus 1–2 MetS components

		CRC				All-Cause Mortality			
**Variable**	**No.**	**Events**	**Adjusted HR** ^**a**^ **(95% CI)**	***P*** **-value**	***P***_***interaction***_	**Events**	**Adjusted HR** ^**a**^ **(95% CI)**	***P*** **-value**	***P***_***interaction***_
Overall	119,843	1,318	**1.28 (1.15–1.43)**	**< 0.001**		10,728	**1.13 (1.08–1.17)**	**< 0.001**	
Age, years					0.731				**< 0.001**
< 65	70,299	526	**1.29 (1.09–1.54)**	**0.004**		3,028	**1.35 (1.25–1.45)**	**< 0.001**	
≥ 65	49,544	792	**1.24 (1.07–1.43)**	**0.003**		7,700	0.99 (0.94–1.04)	0.600	
Gender					0.314				**0.005**
Female	59,862	604	**1.37 (1.16–1.61)**	**< 0.001**		4,886	**1.2 (1.13–1.27)**	**< 0.001**	
Male	59,981	714	**1.22 (1.05–1.42)**	**0.011**		5,842	**1.06 (1.01–1.12)**	**0.024**	
History of CHD					**0.011**				**0.020**
Without	78,844	824	**1.41 (1.23–1.62)**	**< 0.001**		6,261	**1.13 (1.08–1.19)**	**< 0.001**	
With	40,999	494	1.08 (0.90–1.31)	0.398		4,467	**1.13 (1.06–1.20)**	**< 0.001**	
History of CKD					0.587				0.300
Without	115,741	1266	**1.27 (1.14–1.43)**	**< 0.001**		9,998	**1.13 (1.09–1.18)**	**< 0.001**	
With	4,102	52	1.50 (0.86–2.61)	0.151		730	0.97 (0.83–1.13)	0.706	
Aspirin					0.118				0.420
Non-user	83,282	869	**1.35 (1.18–1.54)**	**< 0.001**		5,201	**1.06 (1.00-1.12)**	**0.045**	
User	36,561	449	1.17 (0.97–1.42)	0.105		5,527	**1.24 (1.17–1.31)**	**< 0.001**	
NSAIDs					0.839				**< 0.001**
Non-user	43,008	450	**1.32 (1.09–1.6)**	**0.004**		2,422	1.02 (0.93–1.11)	0.727	
User	76,835	868	**1.27 (1.11–1.45)**	**< 0.001**		8,306	**1.18 (1.13–1.23)**	**< 0.001**	
Statins					0.399				**< 0.001**
Non-user	82,610	956	**1.26 (1.11–1.44)**	**< 0.001**		7,807	1.00 (0.95–1.05)	0.945	
User	37,233	362	**1.31 (1.07–1.62)**	**0.010**		2,921	**1.54 (1.43–1.66)**	**< 0.001**	
Significant values (p < 0.05) between outcome and the stratified covariate are showing in bold. ^a^ Adjusted for age, sex, index year, smoking, alcohol drinking, comorbidities, renal transplantation and concurrent medications, except for the stratified covariate. ^b^ Not available (NA) due to a low sample size.									
Abbreviations: CHD, coronary heart disease; CKD, chronic kidney disease; HR, hazard ratio; NSAID, nonsteroidal anti-inflammatory drugs.									

### Stratified risks for CRC and all-cause mortality

Subjects with 3–4 MetS components had significantly greater risk for CRC in most subgroups compared to those with 1–2 MetS components, except for individuals who had CHD history (aHR, 1.08; 95% CI, 0.90–1.31, p = 0.398), CKD history (aHR, 1.50; 95% CI, 0.86–2.61, p = 0.151), aspirin users (aHR, 1.17; 95% CI, 0.97–1.42, p = 0.105). On the other hand, subjects with 3–4 MetS components also had significantly greater risk for all-cuase mortality in most subgroups, except for the subgroups who aged ≥ 65y, had CKD history, who were NSAIDs non-user or statin non-user (Table 3).

## Discussion

This study investigated the influence of more MetS components compared to fewer MetS components on the risk of CRC and all-cause mortality. The results revealed that subjects with 3–4 MetS components had a significantly higher risk of CRC and all-cause mortality than those with 1–2 components, independent from age, gender, smoking, alcohol drinking, comorbidities, renal transplantation and medications prescribed. Furthermore, the said increased risks were found in most of the subgroups.

Few studies focused on the role of number of MetS components on the risk of CRC and all-cause mortality. A previous systematic review and meta-analysis including 11,462 cancer cases showed an association between MetS and an increased risk of CRC incidence and mortality in both genders [3]. However, the authors concluded that the risk conveyed by the full syndrome was not superior to the sum of its individual components such as higher BMI/waist (RR: 1.19), dysglycemia (RR: 1.29), and higher blood pressure (RR of 1.09) [3]. Another retrospective cohort study included 4,000 persons indicated that MetS was associated with a higher risk of cardiovascular disease (CVD) mortality. However, the authors also mentioned that hypertension, one of MetS components, explained most of the risk. Therefore, although MetS is a risk factor for CVD mortality, it is not beyond the risk from its parts [26]. The present study with a large sample sizes may shed new light on this controversial issue.

An increased risk of CRC in women rather than men was reported in a recent Korean National Cancer Center (KNCC) Community Cohort study including 2,417 men and 4,568 women [4]. However, in a large case-control study with a cohort of 7,558 people in Germany, the absolute risk of CRC in 50-year-old men was higher than that in women (men, 3.5-13.4%; women, 2.5-10.6%) [27]. In this study, 3–4 MetS components led to greater CRC risk and all-cause mortality than 1–2 MetS components among both females and males.

Older age, comorbid CHD, or CKD have been thought as the risk factors of MetS [28–30]. In the stratified analysis of the present study, more MetS components does not show a significant impact on the risk of all-cause mortality among the subgroups older than 65, with CHD, or with CKD than fewer MetS components. These results indicated that the risk posed by more MetS components is modified by age and some comorbidities. The risk discrepancies found may be explained by that older adults were offered routine CRC screening and surveillance with colonoscopy treatment, which attenuated the risk of death posed by more MetS components. A previous study reported that the absolute risk of CRC in 50-year-old patients with colonoscopy treatment was lower than those without colonoscopy [27].

Aspirin has been demonstrated to reduce the risk of CRC and CRC-associated mortality under long-term usage [31, 32]. Furthermore, CRC patients who used aspirin before age 60–70 years and continued to use had a reduced risk of CRC and CRC-associated death [33, 34]. In this study, aspirin users with more MetS components did not have a significantly higher risk of CRC than those with fewer MetS components. Chubak et al. found no substantial effect of aspirin intake on all-cause mortality rate of CRC patients within 10 years of use [31]. Taking together, the long-term interaction between aspirin use and MetS on CRC and mortality still needs to be investigated in the future.

NSAID, such as rofecoxib or celecoxib, were CRC chemoprevention agents associated with a reduced CRC risk or a reduced cumulative incidence of one or more adenomas [35–37]. In this study, more MetS components seemed not increase the risk of all-cause mortality than fewer MetS components among NSAIDs non-user.

Like aspirin, statins are also identified as chemoprevention agents and might be used for average to high-risk population [35]. These agents have been shown to associate with lower risks of CRC, post-colonoscopy colorectal cancer (PCCRC), and mortality [35, 38]. However, the protective effect of statins on CRC is still under debate. A previous case-control study containing 25,811 CRC cases from the System for Development of Primary Care Research (SIDIAP) database revealed no significant decrease of CRC risk related to statin exposure [39]. In this study, more MetS components did not significantly increased the risk of all-cause mortality among statin non-users. Similarly, interactions between statin use and MetS on CRC and mortality still should be continuously evaluated in the future studies.

We did not included metformin usage in the present study. A previous trial has documented the chemoprevention effect of colorectal adenoma or polyps in post-polypectomy patients without diabetes [40]. This also warrants the need for future investigation on the interactions between metformin and MetS on CRC risks.

### Strengths and limitations

The major strengths of the present study was the utilization of a comprehensive national dataset with a large sample. In addition, longitudinal follow-up data allowed us to conduct long-term observations.

Nevertheless, there were limitations in this study. Firstly, the study relied on administrative codes, which the accuracy of could have influence the study results. Secondly, NHIRD lacked the information on waist circumference, thus obesity based on BMI is used instead, which may underestimate the prevalence of MetS. Thirdly, there may be a dose difference between the prescribed medications. Further, it is known that endoscopic removal of colorectal adenomas is important for primary prevention of colorectal cancer, however, lacking data of whether colonoscopy was performed hampered further analysis. In addition, NHIRD lacked some crucial information that may be confounding factors, such as patients’ dietary habits, physical activities, or glycemic control in DM. Although CRC-related mortality would be more informative than overall mortality, such data were not available, and it definitely needs to be addressed in the future.

## Conclusion

Increased risks of CRC and all-cause mortality are found in subjects with more MetS components than fewer components. Further, the increased risks were demonstrated in most subgroups. Knowledge gained from this study may help clinicians on the CRC risk stratification according to individuals’ characteristics, as well as to optimize the strategy of MetS surveillance and control in order to prevent CRC.

## Data Availability

The datasets used during the current study are available from the corresponding author on reasonable request.

## List of abbreviations

MetS: Metabolic syndrome.
CRC: colorectal cancer.
NHIRD: National Health Insurance Research Database.
CHD: coronary heart disease.
CKD: chronic kidney disease.

## References

[1] Esposito K, Chiodini P, Colao A, Lenzi A, Giugliano D (2012). Metabolic syndrome and risk of cancer: a systematic review and meta-analysis. Diabetes Care.

[2] Kassi E, Pervanidou P, Kaltsas G, Chrousos G (2011). Metabolic syndrome: definitions and controversies. BMC Med.

[3] Esposito K, Chiodini P, Capuano A, Bellastella G, Maiorino MI, Rafaniello C (2013). Colorectal cancer association with metabolic syndrome and its components: a systematic review with meta-analysis. Endocrine.

[4] Kim J, Park EY, Park E, Lim MK, Oh JK, Kim B. Metabolic Syndrome and Colorectal Cancer Risk: Results of Propensity Score-Based Analyses in a Community-Based Cohort Study. Int J Environ Res Public Health. 2020; 17(22). 10.3390/ijerph17228687 PMC7700241 33238496

[5] Mozumdar A, Liguori G (2011). Persistent increase of prevalence of metabolic syndrome among U.S. adults: NHANES III to NHANES 1999–2006. Diabetes Care.

[6] Shariq OA, Hanson KT, McKenna NP, Kelley SR, Dozois EJ, Lightner AL (2019). Does Metabolic Syndrome Increase the Risk of Postoperative Complications in Patients Undergoing Colorectal Cancer Surgery?. Dis Colon Rectum.

[7] Alokail MS, Al-Daghri N, Abdulkareem A, Draz HM, Yakout SM, Alnaami AM (2013). Metabolic syndrome biomarkers and early breast cancer in Saudi women: evidence for the presence of a systemic stress response and/or a pre-existing metabolic syndrome-related neoplasia risk?. BMC Cancer.

[8] Russo A, Autelitano M, Bisanti L (2008). Metabolic syndrome and cancer risk. Eur J Cancer.

[9] Arnold M, Sierra MS, Laversanne M, Soerjomataram I, Jemal A, Bray F (2017). Global patterns and trends in colorectal cancer incidence and mortality. Gut.

[10] Siegel RL, Miller KD, Goding Sauer A, Fedewa SA, Butterly LF, Anderson JC (2020). Colorectal cancer statistics, 2020. CA Cancer J Clin.

[11] Bardou M, Barkun AN, Martel M (2013). Obesity and colorectal cancer. Gut.

[12] Bouvard V, Loomis D, Guyton KZ, Grosse Y, Ghissassi FE, Benbrahim-Tallaa L (2015). Carcinogenicity of consumption of red and processed meat. Lancet Oncol.

[13] Chen DZ, Ji FY, Xu QM, Wu XX, Cai C, Zhang LJ (2018). Interaction of smoking and metabolic syndrome in increasing the recurrence risk of colorectal cancer in a Chinese male cohort: a retrospective study. Sci Rep.

[14] Doubeni CA, Major JM, Laiyemo AO, Schootman M, Zauber AG, Hollenbeck AR (2012). Contribution of behavioral risk factors and obesity to socioeconomic differences in colorectal cancer incidence. J Natl Cancer Inst.

[15] He Q, Zhang H, Yao S, Zhu D, Lv D, Cui P (2018). A study on relationship between metabolic syndrome and colorectal cancer. J buon.

[16] Li X, Chen H, Wang G, Feng X, Lyu Z, Wei L, et al. Metabolic Syndrome Components and the Risk of Colorectal Cancer: A Population-Based Prospective Study in Chinese Men. Front Oncol. 2019; 9 1047. 10.3389/fonc.2019.01047 PMC6811600 31681585

[17] Milano A, Bianco MA, Buri L, Cipolletta L, Grossi E, Rotondano G, et al. Metabolic syndrome is a risk factor for colorectal adenoma and cancer: a study in a White population using the harmonized criteria. Therap Adv Gastroenterol. 2019; 12 1756284819867839. 10.1177/1756284819867839 PMC6727097 31523276

[18] Ulaganathan V, Kandiah M, Mohd Shariff Z (2018). A case-control study of the association between metabolic syndrome and colorectal cancer: a comparison of International Diabetes Federation, National Cholesterol Education Program Adults Treatment Panel III, and World Health Organization definitions. J Gastrointest Oncol.

[19] Kabat GC, Kim MY, Stefanick M, Ho GYF, Lane DS, Odegaard AO (2018). Metabolic obesity phenotypes and risk of colorectal cancer in postmenopausal women. Int J Cancer.

[20] Reed M, Patrick C, Croft B, Walde N, Voutsadakis IA (2019). The metabolic syndrome and its components as prognostic factors in metastatic colorectal cancer. Indian J Gastroenterol.

[21] Lin LY, Warren-Gash C, Smeeth L, Chen PC. Data resource profile: the National Health Insurance Research Database (NHIRD). Epidemiol Health. 2018; 40 e2018062. 10.4178/epih.e2018062 PMC6367203 30727703

[22] Raviv NV, Sakhuja S, Schlachter M, Akinyemiju T (2017). Metabolic syndrome and in-hospital outcomes among pancreatic cancer patients. Diabetes Metab Syndr.

[23] Austin PC (2009). Some methods of propensity-score matching had superior performance to others: results of an empirical investigation and Monte Carlo simulations. Biom J.

[24] Austin PC (2008). Assessing balance in measured baseline covariates when using many-to-one matching on the propensity-score. Pharmacoepidemiol Drug Saf.

[25] Austin PC (2013). The performance of different propensity score methods for estimating marginal hazard ratios. Stat Med.

[26] Mazloomzadeh S, Karami Zarandi F, Shoghli A, Dinmohammadi H. Metabolic syndrome, its components and mortality: A population-based study. Med J Islam Repub Iran. 2019; 33 11. 10.34171/mjiri.33.11 PMC6504944 31086790

[27] Carr PR, Weigl K, Edelmann D, Jansen L, Chang-Claude J, Brenner H, et al. Estimation of Absolute Risk of Colorectal Cancer Based on Healthy Lifestyle, Genetic Risk, and Colonoscopy Status in a Population-Based Study. Gastroenterology. 2020; 159(1): 129 – 38.e9. 10.1053/j.gastro.2020.03.016 PMC7387145 32179093

[28] Agarwal S, Shlipak MG, Kramer H, Jain A, Herrington DM. The association of chronic kidney disease and metabolic syndrome with incident cardiovascular events: multiethnic study of atherosclerosis. Cardiol Res Pract. 2012; 2012 806102. 10.1155/2012/806102 PMC3154776 21860804

[29] DeBoer MD, Filipp SL, Musani SK, Sims M, Okusa MD, Gurka M (2018). Metabolic Syndrome Severity and Risk of CKD and Worsened GFR: The Jackson Heart Study. Kidney Blood Press Res.

[30] Vishram JK, Borglykke A, Andreasen AH, Jeppesen J, Ibsen H, Jørgensen T (2014). Impact of age and gender on the prevalence and prognostic importance of the metabolic syndrome and its components in Europeans. The MORGAM Prospective Cohort Project. PLoS One.

[31] Chubak JKA, Buist DSM, Anderson ML, Whitlock EP. Aspirin Use for the Prevention of Colorectal Cancer: An Updated Systematic Evidence Review for the U.S. Preventive Services Task Force [Internet]. Rockville (MD): Agency for Healthcare Research and Quality (US); 2015. Report No.: 15-05228-EF-1. 2015. 26491758

[32] Garcia-Albeniz X, Chan AT. Aspirin for the prevention of colorectal cancer. Best Pract Res Clin Gastroenterol. 2011; 25(4–5): 461 – 72. 10.1016/j.bpg.2011.10.015 PMC3354696 22122763

[33] Guo CG, Ma W, Drew DA, Cao Y, Nguyen LH, Joshi AD (2021). Aspirin Use and Risk of Colorectal Cancer Among Older Adults. JAMA Oncol.

[34] Lin HD, Vora P, Soriano-Gabarró M, Chan KA (2020). Association Between Low-Dose Aspirin Use and Colorectal Cancer Incidence in Taiwan. JAMA Netw Open.

[35] Katona BW, Weiss JM (2020). Chemoprevention of Colorectal Cancer. Gastroenterology.

[36] Mohammed A, Yarla NS, Madka V, Rao CV. Clinically Relevant Anti-Inflammatory Agents for Chemoprevention of Colorectal Cancer: New Perspectives. Int J Mol Sci. 2018; 19(8). 10.3390/ijms19082332 PMC6121559 30096840

[37] Sada O, Ahmed K, Jeldo A, Shafi M (2020). Role of Anti-inflammatory Drugs in the Colorectal Cancer. Hosp Pharm.

[38] Cheung KS, Chen L, Chan EW, Seto WK, Wong ICK, Leung WK (2019). Statins reduce the progression of non-advanced adenomas to colorectal cancer: a postcolonoscopy study in 187 897 patients. Gut.

[39] Ibáñez-Sanz G, Guinó E, Pontes C, Quijada-Manuitt M, de la Peña-Negro LC, Aragón M (2019). Statin use and the risk of colorectal cancer in a population-based electronic health records study. Sci Rep.

[40] Higurashi T, Hosono K, Takahashi H, Komiya Y, Umezawa S, Sakai E (2016). Metformin for chemoprevention of metachronous colorectal adenoma or polyps in post-polypectomy patients without diabetes: a multicentre double-blind, placebo-controlled, randomised phase 3 trial. Lancet Oncol.

